# Effect of storage temperature on lipid oxidation and changes in nutrient contents in peanuts

**DOI:** 10.1002/fsn3.1069

**Published:** 2019-06-11

**Authors:** Kunlun Liu, Ying Liu, Fusheng Chen

**Affiliations:** ^1^ College of Food Science and Technology Henan University of Technology Zhengzhou China

**Keywords:** lipid oxidation, nutrient contents, peanut, storage temperature

## Abstract

Peanut, an important oil crop worldwide, is highly susceptible to oxidative damage during storage due to its high level of fats and unsaturated fatty acids which will affects its nutritional value and agricultural importance. Therefore, it is significantly important to research the physicochemical properties changes of peanuts during storage. Peanuts belong to two varieties were stored at various temperatures (15°C, 25°C, and 35°C) for 320 days. Peroxide value (PV), carbonyl value (CV), and malondialdehyde (MDA) content of oil extracted from peanuts were determined every 80 days to evaluate lipid oxidation degree. Proximate composition (fat, protein, total sugar, moisture, and ash), fatty acid, and amino acid compositions were also assessed. All samples exhibited increased CV and MDA contents during storage. The PV of peanuts increased continuously when stored at 15°C and 25°C, but the PV increased firstly and then decreased sharply when stored at 35°C. Storage significantly affected the contents of lipids, proteins, total sugars, and moisture in peanuts but did not influence the ash content. In general, the fatty acid and amino acid compositions changed significantly during storage at different temperatures. High temperatures lead to a high degree of lipid oxidation and nutrient loss. The results above of this study can provide a theoretical basis for the actual storage and preservation of peanuts.

## 1 INTRODUCTION

Peanuts, one of the four major oil crops, are economically important in many countries such as India, China, and the United States (Baker, Sims, Gorbet, Sanders, & O'Keefe, 2010). Peanuts are rich in nutrients, including fat, protein, sugar, fatty acids, and free amino acids, which are indispensable in human life. Peanuts have gained importance due to their potential in lowering cholesterol, delaying human aging, and preventing cancer (Mora‐Escobedo, Hernández‐Luna, Joaquín‐Torres, Ortiz‐Moreno, & Robles‐Ramírez, 2015). However, peanuts are susceptible to lipid autoxidation because of their high contents of fats and unsaturated fatty acids. Lipid oxidation occurs during storage, and the formation of oxidation products is associated with changes in the flavor and nutrient value of peanuts (Jensen, Danielsen, Bertelsen, Skibsted, & Andersen, 2005). In addition to their nature, the other factors that influence the oxidation of peanuts include humidity, temperature, oxygen, and light (Mexis, Badeka, Riganakos, Karakostas, & Kontominas, 2009).

To maintain the quality of peanuts during storage and reduce economic losses, many researchers have studied the lipid oxidation and oxidative stability of peanuts under different storage conditions. Temperature is one of the most important factors that affect lipid oxidation. Nepote, Mestrallet, and Grosso (2006) and Nepote, Mestrallet, Ryan, Conci, and Grosso (2006) determined the oxidative stability of two kinds of roasted peanuts stored at −15°C, 23°C, and 40°C. Results showed that the peroxide value (PV), TBARS value, oxidized levels, and cardboard flavor increased, whereas the roasted peanutty flavor decreased with increasing storage time and temperature. The latest study of Pęksa, Miedzianka, and Nemś (2018) which stored fresh‐colored potatoes at 2°C and 5°C for 3 and 6 months proved that the total protein content and particularly amino acid content declined with increased storage time. Oxygen concentration and light considerably affected the oxidation rate and thus influenced the product flavor. Jensen et al. (2005) reported that increased oxygen availability and exposure to light accelerated lipid oxidation. Light accounted for the greatest systematic variation in the level of free radicals, whereas oxygen availability exhibited the largest influence on the formation of hexanal. Several studies have focused on the influence of relative humidity on the quality of peanut during storage. For example, Mutegi, Wagacha, Christie, Kimani, and Karanja (2013) demonstrated that the moisture content, physical damage, rancidity, and aflatoxin levels of peanuts significantly changed under different humidity levels. Besides, packaging materials were also an important factor for food preservation (Mexis et al., 2009).

Substantial studies have reported on storage conditions or quality deterioration of peanuts. However, few works have investigated changes in the contents of nutrients, especially amino acid composition, of peanuts during long‐term storage. The present study aimed to investigate lipid oxidation and changes in nutrient contents (especially for amino acid) in peanuts during storage at three different temperatures (low, common, and high temperature).

## 2 MATERIALS AND METHODS

### 2.1 Samples

Two shelled peanut seeds with name of YuHua‐9326 (YH‐9326) and YuHua‐22 (YH‐22) were harvested from Xingyang (Latitude 34°36′N, Longitude 113°09′E), China, during September (average temperature: 15°C‒25°C). The shells were removed manually and stored at −20°C for further treatment.

### 2.2 Experimental design

For storage, peanut seeds were divided into three equal portions (250 g in each) and sealed into cloth bags. Samples in bags were placed individually in a controlled temperature incubator of 15°C, 25°C, and 35°C, keeping humidity of 70%. The samples were collected for further analysis after 0, 80,160, 240, and 320 days of storage, respectively (Figure S1).

### 2.3 Proximate composition analysis

Peanut protein content was determined by the AOAC method 988.05 (AOAC, 1995). Crude fat content was determined by Soxhlet extraction in accordance with AOAC 996.06 (AOAC, 1995). Ash content of peanut was analyzed by AOAC method 923.03 (AOAC, 1995). Moisture was measured by AOAC method 934.01 (AOAC, 1995). Total sugar was determined by measuring the absorbance at 620 nm with a spectrophotometer (722s; Inesa). Water activity was estimated by the water activity meter (Labmaster‐aw Novasina). Samples were placed in a sealed and the rmostatic chamber of water activity meter, when the moisture of samples were diffused balancedly, the response value displayed by the sensor in the measuring instrument was the water activity of the samples.

### 2.4 Lipid oxidation

#### 2.4.1 Peroxide value

The PV of peanut oil was determined by the previous method (Shantha & Decker, 1994). Briefly, 5 ml of a mixed solution of chloroform and methanol was used to dissolve the oil extract from peanut. This mixture was adequately blended with ferrous chloride solution and potassium thiocyanate solution and then incubated for 5 min at room temperature. Absorbance of the solution was measured at 500 nm with a spectrophotometer (722s; Inesa).

#### 2.4.2 Carbonyl value

The carbonyl value (CV) was analyzed by the method described by Liu, Yang, Chen, and Fang (2016). Weighed 0.2 g of the peanut oil sample dissolved in benzene. Each peanut oil sample was then added to 3 ml of trichloroacetic acid solution and 5 ml of 2,4‐dinitrophenylhydrazine and mixed well in a tube. Samples were heated in a water bath at 60°C for 30 min, cooled rapidly and mixed with potassium hydroxide‐methanol solution (10 ml, every 100 ml ethanol‐dissolved 4 g potassium hydroxide). The absorbance of the mixture was determined at 440 nm.

#### 2.4.3 Malondialdehyde content

The malondialdehyde (MDA) content was measured according the method reported by Fan and Thayer (2002). The samples were mixed with 10% chloroacetic acid and centrifuged for 15 min (2800*g*), and the supernatant obtained by centrifugation was the MDA extract of the peanut. The extract was mixed with 0.2% thiobarbital acid and reacted in a boiling bath. The absorbance was measured at 450, 500, and 600 nm with a spectrophotometer (722s; Inesa).

### 2.5 Fatty acid composition

Fatty acid composition was measured according to the method reported by Mexis et al. (2009). Approximately 0.1 g of peanut oil was blended with sodium hydroxide and methanol and then esterified drastically. The samples were placed in a 1.5‐ml bottle and injected in the gas chromatography (GC) (7890A/5975C; Agilent) unit. During injection, the injector was operated in the split mode (1:2 split ratio) at a temperature of 330°C.

### 2.6 Amino acid analysis

Amino acid analysis contains total amino acid analysis and free amino acid analysis. The total amino acid analysis includes the content of amino acids initially present in peanuts and the amino acids formed by the protein hydrolyzed, whereas the free amino acid analysis only includes the content of amino acids initially present in peanuts. Total amino acid composition was determined using an automated amino acid analyzer (S433D; Sykam) after hydrolyzing the defatted peanut flour with 6 M HCl at 110°C for 24 hr (Latif, Pfannstiel, Makkar, & Becker, 2013). Free amino acid composition was determined based on the method reported by Tanimoto, Kawakami, and Morimoto (2013) and modified slightly. The free amino acid was extracted with hydrochloric acid from defatted peanut flour, and the extracted solution was mixed with the salicylic acid (5%) to precipitate the protein before determined using amino acid analyzer.

### 2.7 Statistical analysis

Values were expressed as means ± standard deviations, and measurements were obtained in triplicate. The significant difference was determined at the *p* < 0.05 level for Duncan's multiple range test by SPSS software (version 20.0).

## 3 RESULTS AND DISCUSSION

### 3.1 Lipid oxidation

Lipid oxidation occurs during the storage of peanuts and leads to the development of undesirable flavor and color (Nepote, Mestrallet, & Grosso, 2006; Nepote, Mestrallet, Ryan, et al., 2006). The oxidation reactions first lead to the formation of hydroperoxide, which further forms into secondary oxidation product, such as ketones and aldehydes.

#### 3.1.1 Peroxide value

**Figure 1:**
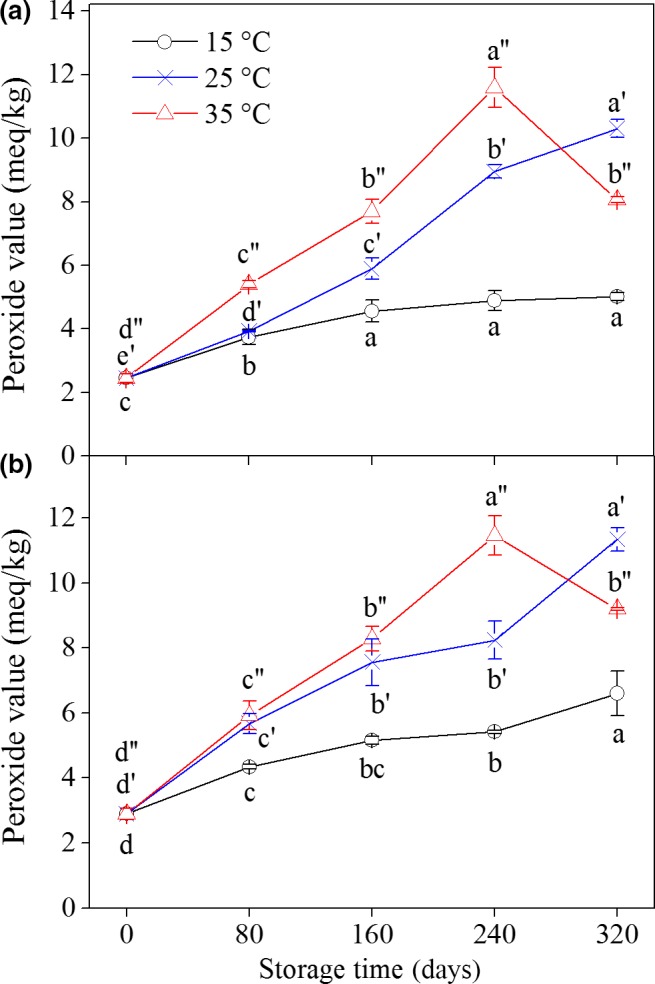
Changes in peroxide value of peanuts during storage at different temperatures. (a): YH‐9326; (b): YH‐22. Each data point represents the mean of three replicate samples. Vertical bars represent the standard errors of means. Values of each peanut cultivar followed by the same letter are not significantly different (*p* ˃ 0.05)

Peroxide value measures the content of hydroperoxides and is often used as an indicator of the primary products of lipid oxidation (Gray, 1978). The change in the PV of two varieties of peanuts during storage is illustrated in Figure 1 and Table S1. The initial PVs of both kinds were maintained at low levels. The PVs of all samples stored at different temperatures increased gradually, except for those stored at 35°C for 320 days. The PV increased more rapidly at high temperatures (25°C and 35°C) than at low temperature (15°C). When stored at 15°C, the PV of YH‐9326 and YH‐22 peanuts was within the acceptable limits (10 meq/kg) to ensure food freshness throughout the storage period. However, the PV was beyond the acceptable limits when stored at 35°C for 240 days. Interestingly, the PV of the two peanut varieties demonstrated a downward trend when stored at 35°C for 320 days. Brannan, Koehler, and Ware (1999) reported that the PV of peanuts stored at 25°C and 63°C increased until week four and then generally declined thereafter. This phenomenon may be due to the fact that the initial steps of lipid oxidation involve chain reactions that form hydroperoxides which are classified as primary lipid oxidation products and could generate secondary lipid oxidation products (Andersen & Skibsted, 2002). Hence, storage temperature and storage time significantly (*p* < 0.05) affected the PV of peanuts, and the former showed more significant effects. Similar results have been reported in other materials. Garcia‐Pascual, Mateos, Carbonell, and Salazar (2003) indicated that PV reached a high level in almond nuts stored at a high temperature for a short period of time. The PV of walnut flour increased gradually when stored at different temperatures for 26 weeks; the hottest storage condition (23°C) led to the highest PV (Vanhanen & Savage, 2006). In addition, the PVs of walnuts were 15 and 32 meq/kg after storage for 12 months at 4°C and 20°C, respectively (Mexis et al., 2009).

#### 3.1.2 Malondialdehyde content

**Figure 2:**
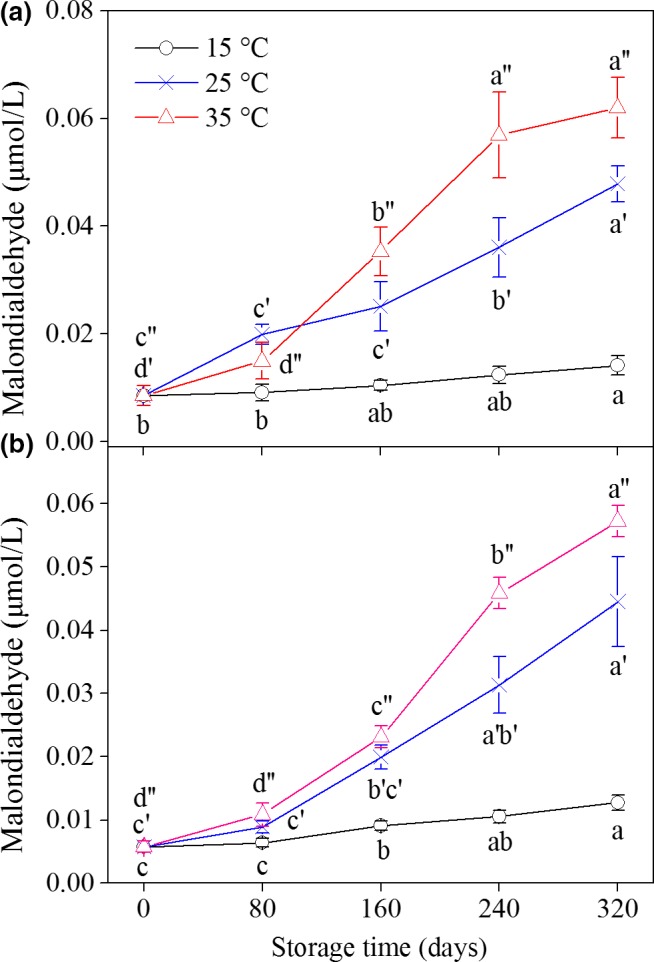
Changes in malondialdehyde content of peanuts during storage at different temperatures. (a): YH‐9326; (b): YH‐22. Each data point represents the mean of three replicate samples. Vertical bars represent the standard errors of means. Values of each peanut cultivar followed by the same letter are not significantly different (*p* ˃ 0.05)

Malondialdehyde is a small‐molecule substance formed from hydroperoxide, which is the initial reaction product of polyunsaturated fatty acids with oxygen. MDA is usually regarded as the final product of lipid oxidation and responsible for the development of an objectionable odor (Goulas & Kontominas, 2007). Figure 2 shows the influence of storage temperature on MDA content in different kinds of peanuts. The MDA contents in all samples changed significantly (*p* < 0.05) with increasing storage temperature and prolonged storage time. The MDA content increased slowly throughout the entire storage period at 15°C. Meanwhile, the content increased rapidly when stored at 25°C and/or 35°C. For example, after 320 days of storage, the MDA content in YH‐9326 increased by 3.4 and 4.4 times after storage at 25°C and 35°C, respectively, relative to that stored at 15°C. Temba, Njobeh, and Kayitesi (2017) stored groundnut flour at room temperature for 3 months and found that the thiobarbituric acid value, which reflects the MDA content, increased significantly with increasing storage period. Similarly, Mexis et al. (2009) proved that storage of walnuts at high temperatures resulted in higher MDA content than storage at low temperatures.

#### 3.1.3 Carbonyl value

**Figure 3:**
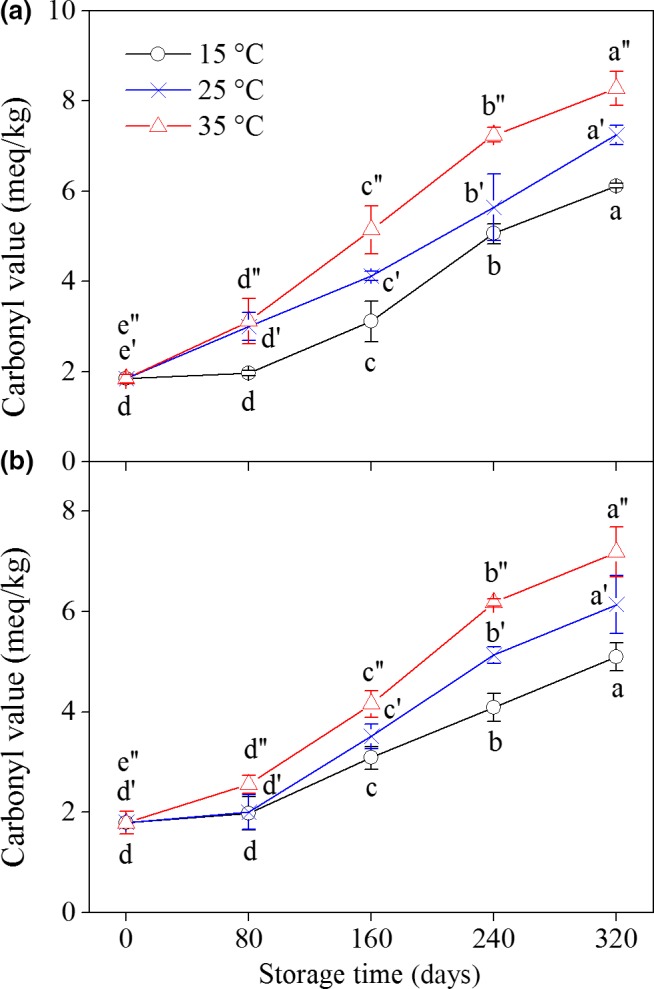
Changes in carbonyl value of peanuts during storage at different temperatures. (a): YH‐9326; (b): YH‐22. Each data point represents the mean of three replicate samples. Vertical bars represent the standard errors of means. Values of each peanut cultivar followed by the same letter are not significantly different (*p* ˃ 0.05)

Carbonyl compounds are a general term for aldehydes, ketones, and carboxylic acid. CV is a suitable indicator for evaluating lipid oxidation. The CVs of each peanut type are presented in Figure 3. The CVs of YH‐9326 and YH‐22 peanuts stored at different temperatures increased with increasing temperature and storage time. Interestingly, peanuts exhibited a smaller increase in the speed of CV before 80 days and a greater speed in the later period (160–320 days). Moreover, high storage temperatures led to the high CV of these two kinds of peanuts.

According to the results of PV, MDA, and CV, peanut lipids oxidized to various levels across the storage time period and different temperatures. The oxidation degrees increased significantly (*p* < 0.05) with increasing storage time and temperature, indicating that heat accelerated lipid oxidation. During oxidation, lipids mainly form intermediates, such as hydroperoxides, in the early stage of storage and then form other molecule products (e.g., aldehydes and ketones) (Abegaz, Kerr, & Koehler, 2004). This phenomenon may be the reason why the PV of the samples increased firstly and then decreased throughout the storage period under 35°C. Meanwhile, the CV and MDA contents in the peanut samples demonstrated continuous increase during storage. A number of articles have reported that sensory attributes and acceptability were positively associated with PV, CV, and MDA. Hence, the storage period of peanuts can be controlled to meet the consumers’ requirement, thereby reducing unnecessary economic losses (Nepote, Olmedo, Mestrallet, & Grosso, 2010).

### 3.2 Change in nutrient contents

#### 3.2.1 Proximate composition

The proximate compositions (lipid, protein, total sugar, moisture, and ash) of peanuts YH‐9326 and YH‐22 stored at different temperatures are presented in Table 1. The lipid and protein contents in both varieties of peanuts showed no significant changes during 320 days of storage at 15°C and 25°C, but decreased significantly (*p* < 0.05) in YH‐9326 peanuts stored at 35°C for prolonged duration. At the same storage time, no significant effects were observed on lipid and protein contents among different temperatures. Within the same storage temperature, the storage time (0–320) showed no significant effects on the total sugar contents in peanuts, except for YH‐9326 stored at 35°C. But the storage temperature significantly influenced the changes in the total sugar content. For example, after 320 days storage, the total sugar contents, for YH‐9326, declined from 13.27% to 12.42% when stored at 15°C and 35°C, respectively. This phenomenon may be attributed to the fact that reducing sugars participate in nonenzymatic glycosylation and Maillard browning with amino residues in the protein (Wettlaufer & Leopold, 1991). For YH‐9326 and YH‐22 peanuts, the moisture content increased during the entire storage period, indicating that the peanuts absorbed water from the environment. As expected, the moisture content of peanuts stored at 35°C was lower than those of peanuts stored at 15°C and 25°C. The ash content in all peanut samples remained stable during storage, and no significant changes were found. Scholars have studied changes in the proximate composition of peanuts and other materials during storage. Mutegi et al. (2013) reported that the moisture content in peanuts significantly decreased after 4 months of storage. Garcia‐Pascual et al. (2003) evaluated the fat contents in four varieties (Marcona, Desmayo, Planeta, and Nonpareil) of almond during storage, and the results showed that the fat contents significantly declined compared with the initial values, except for Marcona. Hou and Chang (2004) indicated that the lipid content in soybeans increased, the sugar content decreased, and the protein content changed significantly at the late stages of storage. Overall, whether our results are similar to others or not, the proximate composition of peanuts did significantly change throughout the storage period and even interacted with one another. The differences in the results of our research and previous works may be due to the differences in the materials used and storage conditions. Moreover, different storage temperatures showed varied influences on the proximate composition of peanuts.

**Table 1:** Changes in proximate composition of peanuts YH‐9326 and YH‐22 under various storage temperature conditions

Peanut variety	Temperature (°C)	Storage time (day)	Lipid (%)	Protein (%)	Total sugar (%)	Moisture content (%)	Ash (%)
YH‐9326	15	0	42.47 ± 0.24^a^	24.21 ± 0.01^a^	13.27 ± 0.51^a^	3.65 ± 0.05^e^	2.38 ± 0.01^a^
		80	42.37 ± 0.15^aA^	24.11 ± 0.28^aA^	13.32 ± 0.28^aA^	5.49 ± 0.03^dC^	2.38 ± 0.06^aA^
		160	42.55 ± 0.03^aA^	24.29 ± 0.47^aA^	13.34 ± 0.01^aA^	6.72 ± 0.01^cA^	2.38 ± 0.04^aA^
		240	42.57 ± 0.25^aA^	24.31 ± 0.70^aA^	13.27 ± 0.20^aA^	7.29 ± 0.01^bA^	2.37 ± 0.01^aA^
		320	42.37 ± 0.16^aA^	24.11 ± 0.39^aA^	13.23 ± 0.01^aA^	7.63 ± 0.00^aA^	2.39 ± 0.01^aA^
	25	0	42.47 ± 0.24^a^	24.21 ± 0.01^a^	13.27 ± 0.51^a^	3.65 ± 0.05^b^	2.38 ± 0.01^a^
		80	42.55 ± 0.13^aA^	24.29 ± 0.21^aA^	13.12 ± 0.24^aA^	6.47 ± 0.00^aA^	2.39 ± 0.04^aA^
		160	42.57 ± 0.42^aA^	24.31 ± 0.73^aA^	13.03 ± 0.18^aAB^	6.61 ± 0.91^aA^	2.38 ± 0.06^aA^
		240	42.32 ± 0.07^aA^	24.06 ± 0.51^aA^	12.61 ± 0.33^aB^	7.23 ± 0.04^aA^	2.37 ± 0.01^aA^
		320	42.56 ± 0.22^aA^	24.30 ± 0.23^aA^	12.63 ± 0.18^aB^	7.16 ± 0.21^aB^	2.37 ± 0.03^aA^
	35	0	42.47 ± 0.24^a^	24.21 ± 0.01^a^	13.27 ± 0.51^a^	3.65 ± 0.05^b^	2.38 ± 0.01^a^
		80	42.28 ± 0.43^aA^	24.02 ± 0.89^aA^	13.04 ± 0.11^abA^	5.99 ± 0.00^aB^	2.36 ± 0.08^aA^
		160	42.25 ± 0.02^aA^	23.99 ± 0.23^aA^	12.61 ± 0.26^abB^	6.69 ± 0.71^aA^	2.36 ± 0.10^aA^
		240	41.37 ± 0.26^bB^	23.11 ± 0.34^bB^	12.56 ± 0.14^abB^	6.71 ± 0.05^aB^	2.36 ± 0.06^aA^
		320	41.47 ± 0.31^bB^	23.21 ± 0.21^bB^	12.42 ± 0.07^bB^	6.46 ± 0.14^aC^	2.37 ± 0.13^aA^
YH‐22	15	0	40.96 ± 0.18^a^	23.77 ± 0.08^a^	14.04 ± 0.49^a^	4.05 ± 0.09^c^	2.54 ± 0.16^a^
		80	40.96 ± 0.35^aA^	23.77 ± 0.31^aA^	14.04 ± 0.04^aA^	5.65 ± 0.12^bB^	2.55 ± 0.01^aA^
		160	40.88 ± 0.05^aA^	23.69 ± 0.78^aA^	14.10 ± 0.31^aA^	7.70 ± 0.06^aA^	2.51 ± 0.07^aA^
		240	40.77 ± 0.63^aA^	23.57 ± 0.07^aA^	14.02 ± 0.19^aA^	7.53 ± 0.04^aA^	2.49 ± 0.08^aA^
		320	40.97 ± 0.32^aA^	23.77 ± 0.29^aA^	13.92 ± 0.05^aA^	7.65 ± 0.04^aA^	2.51 ± 0.14^aA^
	25	0	40.96 ± 0.18^a^	23.77 ± 0.08^a^	14.04 ± 0.49^a^	4.05 ± 0.09^d^	2.54 ± 0.16^a^
		80	40.62 ± 0.21^aA^	23.42 ± 0.47^aA^	13.97 ± 0.22^aA^	6.29 ± 0.01^cA^	2.54 ± 0.01^aA^
		160	40.76 ± 0.47^aA^	23.56 ± 0.16^aA^	13.89 ± 0.14^aA^	7.11 ± 0.01^bB^	2.52 ± 0.04^aA^
		240	40.53 ± 0.24^aA^	23.33 ± 0.36^aA^	13.52 ± 0.14^aB^	7.48 ± 0.13^aA^	2.54 ± 0.16^aA^
		320	40.88 ± 0.28^aA^	23.69 ± 0.42^aA^	13.54 ± 0.08^aB^	7.36 ± 0.03^aB^	2.54 ± 0.18^aA^
	35	0	40.96 ± 0.18^a^	23.77 ± 0.08^a^	14.04 ± 0.49^a^	4.05 ± 0.09^e^	2.54 ± 0.16^a^
		80	40.82 ± 0.72^aA^	24.63 ± 0.66^aA^	13.59 ± 0.38^aA^	5.85 ± 0.12^dB^	2.50 ± 0.08^aA^
		160	40.15 ± 0.38^aA^	22.96 ± 0.16^aA^	13.45 ± 0.14^aA^	7.04 ± 0.01^aB^	2.58 ± 0.06^aA^
		240	40.21 ± 0.17^aA^	23.01 ± 0.42^aA^	13.43 ± 0.04^aB^	6.82 ± 0.03^bB^	2.53 ± 0.01^aA^
		320	40.02 ± 0.34^aA^	22.83 ± 0.48^aA^	13.34 ± 0.17^aB^	6.34 ± 0.06^cC^	2.54 ± 0.04^aA^

Note

Data are expressed as mean ± *SD* with three replications. Different lowercase letters in the same line of the same peanut variety and under the same storage temperature indicate statistically significant different values (*p* < 0.05). Different capital letters in the same line of the same peanut variety and at the same storage time indicate statistically significant different values (*p* < 0.05).

#### 3.2.2 Fatty acid composition

Table 2 shows the changes in peanut fatty acid composition during storage at different temperatures (Supporting Information are given in Figures S2–S6). The fatty acid composition of peanuts (YH‐9326) was dominated by unsaturated fatty acids (80.29%), which plays an important role in our daily life. Oleic acid (41.28%) and linoleic acid (37.14%) were found to be the most abundant. The changes in different fatty acids varied at three storage temperatures. No significant change in the arachidic acid (20:0) content was observed throughout the storage period. The oleic acid (18:1) content steadily increased with increasing storage temperature and duration. Meanwhile, the contents of linoleic acid (18:2), linolenic acid (18:3), and behenic acid (22:0) decreased. In addition, long‐term storage led to changes in the total unsaturated fatty acid content. These results suggest that storage temperature and time affected the fatty acid composition of peanuts possibly because of the oxidation of triglycerides and free fatty acids. The contents of free fatty acid were increased by the oxidative decomposition of triglycerides, but decreased by oxidative hydrogenation of the free fatty acids. Similar results were reported by Li et al. (2014), who analyzed changes in the fatty acid content in the eight kinds of vegetable oils during storage under accelerated oxidation conditions. The contents of polyunsaturated fatty acids decreased, whereas those of saturated and monounsaturated fatty acids increased. A previous research (Li et al., 2014) clarified that this phenomenon may be due to the unsaturated fatty acids that are susceptible to oxidation. This result is consistent with those reported by Chávezservín, Castellote, Martín, Chifré, and LópezSabater (2009). However, Zwarts, Savage, and Mcneil (1999) reported changes in fatty acid profiles but did not detect consistent decrease in the polyunsaturated profiles in every cultivar when stored under standard commercial conditions. The oleic/linoleic ratio (O/L) is an index of stability to oxidative damage during refining and storage; high ratios are associated with long shelf life (Mora‐Escobedo et al., 2015). No significant change was observed in the O/L ratio during storage at different temperatures in our study.

**Table 2:** Fatty acid composition of peanut YH‐9326 during storage under different temperatures

Temperature (°C)	Stored time (day)	Palmitic acid	Stearic acid	Oleic acid	Linoleic acid	Linolenic acid	Arachidic acid	Behenic acid	Osenic acid	Saturated fatty acid	Unsaturated fatty acid	O/L
15	0	11.78 ± 0.00^ab^	3.70 ± 0.01^a^	41.28 ± 0.00^b^	37.14 ± 0.04^a^	0.70 ± 0.03^a^	1.60 ± 0.01^a^	2.58 ± 0.01^a^	1.17 ± 0.02^a^	19.67 ± 0.03^a^	80.29 ± 0.10^a^	1.11 ± 0.09^a^
	80	11.89 ± 0.17^abA^	3.66 ± 0.19^aA^	41.11 ± 0.02^bB^	37.48 ± 0.10^aA^	0.67 ± 0.07^aA^	1.60 ± 0.05^aA^	2.56 ± 0.08^abA^	1.04 ± 0.04^bA^	19.70 ± 0.49^aA^	80.30 ± 0.24^aB^	1.10 ± 0.10^aA^
	160	11.96 ± 0.07^aA^	3.90 ± 0.16^aA^	42.34 ± 0.20^aB^	36.03 ± 0.26^bA^	0.65 ± 0.03^aA^	1.60 ± 0.01^aA^	2.48 ± 0.09^abA^	1.04 ± 0.06^bA^	19.95 ± 0.14^aA^	80.06 ± 0.43^aA^	1.18 ± 0.00^aA^
	240	11.63 ± 0.07^bA^	3.81 ± 0.01^aA^	42.69 ± 0.24^aA^	36.06 ± 0.36^bA^	0.69 ± 0.05^aA^	1.58 ± 0.00^aA^	2.42 ± 0.05^bA^	1.08 ± 0.02^abA^	19.44 ± 0.11^aA^	80.53 ± 0.05^aA^	1.18 ± 0.02^aA^
	320	11.95 ± 0.10^aA^	3.81 ± 0.02^aA^	42.70 ± 0.27^aB^	35.99 ± 0.14^bA^	0.63 ± 0.01^aA^	1.58 ± 0.08^aA^	2.43 ± 0.01^abA^	1.09 ± 0.03^abA^	19.76 ± 0.01^aA^	80.06 ± 0.11^aC^	1.19 ± 0.01^aA^
25	0	11.78 ± 0.00^a^	3.70 ± 0.01^a^	41.28 ± 0.00^d^	37.14 ± 0.04^a^	0.70 ± 0.03^a^	1.60 ± 0.01^a^	2.58 ± 0.01^a^	1.17 ± 0.02^a^	19.67 ± 0.03^a^	80.29 ± 0.10^bc^	1.11 ± 0.09^a^
	80	11.91 ± 0.07^aA^	3.65 ± 0.09^aA^	41.91 ± 0.16^cAB^	37.36 ± 0.04^aAB^	0.68 ± 0.00^abA^	1.60 ± 0.11^aA^	2.54 ± 0.07^abA^	1.08 ± 0.04^bA^	19.70 ± 0.34^aA^	81.02 ± 0.07^aAB^	1.12 ± 0.01^aAB^
	160	11.78 ± 0.46^aA^	3.82 ± 0.13^aA^	42.97 ± 0.11^aA^	35.92 ± 0.01^bA^	0.68 ± 0.00^abA^	1.61 ± 0.01^aA^	2.41 ± 0.05^cA^	1.07 ± 0.06^bA^	19.61 ± 0.39^aA^	80.63 ± 0.17^abA^	1.20 ± 0.03^abAB^
	240	11.70 ± 0.07^aA^	3.80 ± 0.14^aA^	42.96 ± 0.15^aA^	35.77 ± 0.21^bA^	0.66 ± 0.00^bA^	1.60 ± 0.10^aA^	2.45 ± 0.05^bcA^	1.06 ± 0.01^bA^	19.55 ± 0.36^aA^	80.45 ± 0.38^bcA^	1.20 ± 0.06^aA^
	320	12.11 ± 0.14^aA^	3.94 ± 0.22^aA^	42.44 ± 0.17^bB^	35.85 ± 0.15^bA^	0.67 ± 0.01^abA^	1.60 ± 0.00^aA^	2.44 ± 0.01^bcA^	1.09 ± 0.00^abA^	20.09 ± 0.37^aA^	80.41 ± 0.03^cB^	1.18 ± 0.01^aA^
35	0	11.78 ± 0.00^a^	3.70 ± 0.01^b^	41.28 ± 0.00^b^	37.14 ± 0.04^ab^	0.70 ± 0.03^a^	1.60 ± 0.01^a^	2.58 ± 0.01^a^	1.17 ± 0.02^a^	19.67 ± 0.03^a^	80.29 ± 0.10^b^	1.11 ± 0.09^a^
	80	12.00 ± 0.15^aA^	3.68 ± 0.12^bA^	42.90 ± 0.57^aA^	37.10 ± 0.12^aB^	0.69 ± 0.01^aA^	1.65 ± 0.04^aA^	2.50 ± 0.14^abA^	1.12 ± 0.16^aA^	19.82 ± 0.44^aA^	81.81 ± 0.59^aA^	1.16 ± 0.02^aA^
	160	11.84 ± 0.55^aA^	3.76 ± 0.06^abA^	43.07 ± 0.03^aA^	35.69 ± 0.43^abA^	0.65 ± 0.01^abA^	1.61 ± 0.00^aA^	2.41 ± 0.01^abA^	1.02 ± 0.15^aA^	19.62 ± 0.49^aA^	80.43 ± 0.30^bA^	1.21 ± 0.10^aA^
	240	11.71 ± 0.01^aA^	3.89 ± 0.07^abA^	43.01 ± 033^aA^	35.73 ± 0.29^bA^	0.63 ± 0.02^bA^	1.58 ± 0.00^aA^	2.41 ± 0.00^abA^	1.01 ± 0.15^aA^	19.59 ± 0.09^aA^	80.37 ± 0.13^bA^	1.20 ± 0.02^aA^
	320	12.15 ± 0.19^aA^	3.96 ± 0.15^aA^	43.33 ± 0.04^aA^	35.75 ± 0.08^bA^	0.62 ± 0.03^bA^	1.59 ± 0.01^aA^	2.40 ± 0.05^bA^	1.06 ± 0.05^aA^	20.10 ± 0.28^aA^	80.76 ± 0.14^bA^	1.21 ± 0.13^aA^

Note

Data are expressed as mean ± *SD* with three replications. Different lowercase letters in the same line and under the same storage temperature indicate statistically significant different values (*p* < 0.05). Different capital letters in the same line and at the same storage time indicate statistically significant different values (*p* < 0.05).

Abbreviation: O/L, ratio of oleic acid to linoleic acid.

#### 3.2.3 Amino acid profile analysis

Amino acids, especially essential amino acids (EAAs), are important nutrients in peanuts. The total amino acid and free amino acid compositions are shown in Tables 3 and 4. The total amino acid composition of peanuts changed significantly during storage, but no consistent tendency was found (Table 3). The content of aspartic acid, glutamic acid, glycine, leucine, tyrosine, and arginine significantly increased with the increasing storage temperature and time. However, the contents of alanine and lysine decreased significantly with increasing storage temperature. Moreover, no significant change was observed in the histidine content throughout the storage duration. Lysine, which is generally deficient in peanuts, has been recognized as the first limiting amino acid of peanuts (Hamaker et al., 1992). The levels of cystine and methionine are lower than the lysine level because the former two were partially destroyed by hydrochloric acid during hydrolysis. Nevertheless, the free amino acid contents in the samples significantly decreased during storage compared with the initial value (Table 4). For example, the aspartic acid of peanut decreased almost by 4 times from 0.55 to 0.13 mg/g, and serine decreased from 0.47 to 0.27 mg/g after stored at 35°C for 320 days. In addition, essential amino acids, which are an important nutrient content, decreased significantly from 0.89 to 0.39 mg/g after storage at 35°C for 320 days. To summarize, most of total amino acid contents in YH‐9326 peanuts increased or did not change during storage. Meanwhile, the free amino acid content decreased continuously. Hence, peanut proteins may oxidize and decompose during storage, resulting in increased partial amino acid contents in the total amino acid analysis. A significant loss of free amino acids, especially essential amino acids, was observed during storage, leading to damage in nutrients of peanuts. Higher storage temperature led to greater losses. Pattee, Young, and Giesbrecht (1981) determined individual free amino acid contents in peanuts (stored at 4°C for 9 months) and found significant linear and/or quadratic changes in 15 of the 18 individual amino acids over storage. Different results were reported by Srivastava, Srivastava, Kumar, and Sinha (2013), who found that the total amino acid content significantly decreased in the *Jatrophacurcas *L. seeds after storage for 1 year. Pęksa et al. (2018) reported that the sum of all amino acid contents and that of all EAA amino acids in potatoes declined during storage. Thus far, few studies have investigated changes in the amino acid composition of peanuts during storage and the underlying mechanism. Further research must be conducted to understand the composition and structure of peanut proteins and/or amino acids during storage.

**Table 3:** Total amino acid analysis of peanut YH‐9326 stored at different temperatures

Amino acid %	15°C			25°C			35°C		
	0 day	160 days	320 days	0 day	160 days	320 days	0 day	160 days	320 days
Asp	5.64 ± 0.01^a^	5.67 ± 0.03^aB^	5.68 ± 0.03^aA^	5.64 ± 0.01^b^	5.88 ± 0.07^aA^	5.94 ± 0.05^aA^	5.64 ± 0.01^b^	6.05 ± 0.02^aA^	5.77 ± 0.11^bA^
Thr	1.00 ± 0.01^a^	1.01 ± 0.04^aA^	1.01 ± 0.08^aA^	1.00 ± 0.01^b^	1.06 ± 0.01^aA^	1.06 ± 0.01^aA^	1.00 ± 0.01^b^	1.06 ± 0.03^aA^	1.01 ± 0.01^abA^
Ser	2.10 ± 0.01^a^	2.17 ± 0.10^aA^	2.14 ± 0.06^aA^	2.10 ± 0.01^b^	2.27 ± 0.07^aA^	2.22 ± 0.02^abA^	2.10 ± 0.01^a^	2.28 ± 0.10^aA^	2.15 ± 0.01^aA^
Glu	10.67 ± 0.04^a^	10.54 ± 0.08^aA^	10.69 ± 0.04^aC^	10.67 ± 0.04^a^	11.05 ± 0.53^aA^	11.01 ± 0.00^aA^	10.67 ± 0.04^c^	11.44 ± 0.00^aA^	10.88 ± 0.00^bB^
Gly	2.88 ± 0.01^a^	2.91 ± 0.04^aB^	2.79 ± 0.03^aB^	2.88 ± 0.01^a^	2.92 ± 0.07^aAB^	3.09 ± 0.11^aA^	2.88 ± 0.01^c^	3.11 ± 0.01^aA^	2.95 ± 0.01^bAB^
Ala	1.89 ± 0.10^a^	1.92 ± 0.02^aA^	1.96 ± 0.00^aB^	1.89 ± 0.10^a^	2.00 ± 0.20^aA^	2.01 ± 0.01^aA^	1.89 ± 0.10^a^	2.03 ± 0.00^aA^	1.92 ± 0.00^aC^
Cys	0.03 ± 0.03^a^	0.03 ± 0.03^aA^	0.03 ± 0.00^aB^	0.03 ± 0.03^a^	0.03 ± 0.01^aA^	0.03 ± 0.01^aA^	0.03 ± 0.03^a^	0.03 ± 0.01^aA^	0.03 ± 0.00^aB^
Val	1.76 ± 0.07^a^	1.65 ± 0.00^aA^	1.66 ± 0.06^aB^	1.76 ± 0.07^a^	1.70 ± 0.05^aA^	1.86 ± 0.01^aA^	1.76 ± 0.07^a^	1.75 ± 0.02^aA^	1.69 ± 0.00^aB^
Met	0.52 ± 0.04^a^	0.56 ± 0.00^aA^	0.47 ± 0.04^aA^	0.52 ± 0.04^a^	0.46 ± 0.00^aB^	0.50 ± 0.04^aA^	0.52 ± 0.04^a^	0.45 ± 0.01^aB^	0.46 ± 0.03^aA^
Ile	1.84 ± 0.08^a^	1.82 ± 0.02^aA^	1.83 ± 0.01^aB^	1.84 ± 0.08^a^	1.89 ± 0.09^aA^	1.93 ± 0.02^aA^	1.84 ± 0.08^a^	1.95 ± 0.02^aA^	1.89 ± 0.01^aAB^
Leu	3.19 ± 0.00^a^	3.18 ± 0.02^aC^	3.20 ± 0.01^aC^	3.19 ± 0.00^b^	3.30 ± 0.01^aB^	3.36 ± 0.02^aA^	3.19 ± 0.00^c^	3.44 ± 0.01^aA^	3.28 ± 0.01^bB^
Tyr	1.66 ± 0.10^a^	1.61 ± 0.04^aA^	1.62 ± 0.01^aB^	1.66 ± 0.10^a^	1.68 ± 0.10^aA^	1.72 ± 0.01^aA^	1.66 ± 0.10^a^	1.74 ± 0.02^aA^	1.64 ± 0.03^aB^
Phe	2.45 ± 0.01^a^	2.34 ± 0.02^bB^	2.35 ± 0.01^bC^	2.45 ± 0.01^c^	2.50 ± 0.00^bA^	2.57 ± 0.00^aA^	2.45 ± 0.01^a^	2.54 ± 0.06^aA^	2.45 ± 0.00^aB^
His	1.66 ± 0.19^a^	1.25 ± 0.02^aA^	1.35 ± 0.01^aA^	1.66 ± 0.19^a^	1.28 ± 0.04^aA^	1.32 ± 0.04^aA^	1.66 ± 0.19^a^	1.31 ± 0.02^aA^	1.35 ± 0.02^aA^
Lys	1.52 ± 0.13^a^	1.51 ± 0.08^aA^	1.54 ± 0.01^aAB^	1.52 ± 0.13^a^	1.58 ± 0.08^aA^	1.62 ± 0.02^aA^	1.52 ± 0.13^a^	1.59 ± 0.04^aA^	1.53 ± 0.03^aB^
Arg	5.16 ± 0.13^a^	5.26 ± 0.04^aB^	5.35 ± 0.01^aA^	5.16 ± 0.13^b^	5.55 ± 0.04^aA^	5.36 ± 0.01^abA^	5.16 ± 0.13^b^	5.58 ± 0.06^aA^	5.37 ± 0.01^abA^
Pro	1.56 ± 0.06^a^	1.45 ± 0.01^aC^	1.50 ± 0.01^aB^	1.56 ± 0.06^a^	1.54 ± 0.00^aB^	1.64 ± 0.01^aA^	1.56 ± 0.06^a^	1.60 ± 0.02^aA^	1.51 ± 0.01^aB^
TAA	45.47 ± 1.03^a^	44.87 ± 0.54^aB^	45.16 ± 0.36^aB^	45.47 ± 1.03^a^	46.69 ± 0.83^aAB^	47.24 ± 0.30^aA^	45.47 ± 1.03^a^	47.96 ± 0.60^aA^	45.87 ± 0.09^aB^

Note

Data are expressed as mean ± *SD* with three replications. Different lowercase letters in the same row and under same storage temperature indicate statistically significant different values (*p* < 0.05). Different capital letters in the same row and at the same storage time indicate statistically significant different values (*p* < 0.05).

Abbreviation: TAA, total amino acids.

**Table 4:** Free amino acid analysis of peanut YH‐9326 stored at different temperatures

Amino acid (mg/g)	15°C			25°C			35°C		
	0 day	160 days	320 days	0 day	160 days	320 days	0 day	160 days	320 days
Asp	0.55 ± 0.00^a^	0.55 ± 0.00^aA^	0.27 ± 0.01^bA^	0.55 ± 0.00^a^	0.56 ± 0.01^aA^	0.21 ± 0.01^bB^	0.55 ± 0.00^a^	0.42 ± 0.00^bB^	0.13 ± 0.01^cC^
Thr	0.06 ± 0.00^a^	0.06 ± 0.00^aA^	0.05 ± 0.01^aA^	0.06 ± 0.00^a^	0.05 ± 0.01^aA^	0.04 ± 0.01^aA^	0.06 ± 0.00^a^	0.06 ± 0.01^abA^	0.04 ± 0.01^bA^
Ser	0.47 ± 0.01^a^	0.46 ± 0.02^aA^	0.38 ± 0.01^bA^	0.47 ± 0.01^a^	0.45 ± 0.00^aA^	0.30 ± 0.02^bA^	0.47 ± 0.01^a^	0.39 ± 0.01^bB^	0.27 ± 0.00^cB^
Glu	1.99 ± 0.12^a^	2.00 ± 0.00^aA^	1.21 ± 0.03^bA^	1.99 ± 0.12^a^	1.56 ± 0.07^bB^	1.13 ± 0.10^cB^	1.19 ± 0.12^a^	0.99 ± 0.14^bC^	0.91 ± 0.01^bC^
Gly	0.37 ± 0.03^a^	0.38 ± 0.02^aA^	0.13 ± 0.02^bA^	0.37 ± 0.03^a^	0.33 ± 0.01^aAB^	0.11 ± 0.00^bAB^	0.37 ± 0.03^a^	0.29 ± 0.04^aB^	0.06 ± 0.01^bB^
Ala	0.35 ± 0.00^a^	0.35 ± 0.03^aA^	0.25 ± 0.00^bA^	0.35 ± 0.0^a^	0.35 ± 0.01^aA^	0.19 ± 0.01^bA^	0.35 ± 0.0^a^	0.32 ± 0.00^bA^	0.15 ± 0.00^cA^
Cys	0.21 ± 0.01^a^	0.21 ± 0.01^aA^	0.19 ± 0.00^aA^	0.21 ± 0.01^a^	0.21 ± 0.01^aA^	0.15 ± 0.00^bA^	0.21 ± 0.01^a^	0.20 ± 0.02^aA^	0.14 ± 0.01^bA^
Val	0.15 ± 0.01^a^	0.15 ± 0.00^aA^	0.09 ± 0.01^bA^	0.15 ± 0.01^a^	0.15 ± 0.01^aA^	0.08 ± 0.01^bA^	0.15 ± 0.01^a^	0.10 ± 0.02^bB^	0.07 ± 0.01^bB^
Met	0.04 ± 0.00^a^	0.05 ± 0.01^aA^	0.03 ± 0.00^aA^	0.04 ± 0.00^a^	0.04 ± 0.00^aA^	0.03 ± 0.00^bA^	0.04 ± 0.00^a^	0.04 ± 0.01^aA^	0.02 ± 0.00^bA^
Ile	0.07 ± 0.01^a^	0.07 ± 0.01^aA^	0.05 ± 0.01^bA^	0.07 ± 0.01^a^	0.07 ± 0.00^aA^	0.04 ± 0.00^bA^	0.07 ± 0.01^a^	0.05 ± 0.01^bB^	0.03 ± 0.00^cB^
Leu	0.09 ± 0.01^a^	0.09 ± 0.00^aA^	0.04 ± 0.01^bA^	0.09 ± 0.01^a^	0.08 ± 0.00^aB^	0.04 ± 0.00^bB^	0.09 ± 0.01^a^	0.05 ± 0.00^bC^	0.03 ± 0.00^bC^
Tyr	0.19 ± 0.03^a^	0.19 ± 0.00^aA^	0.10 ± 0.01^bA^	0.19 ± 0.03^a^	0.16 ± 0.01^aB^	0.08 ± 0.00^bB^	0.19 ± 0.03^a^	0.11 ± 0.01^bC^	0.05 ± 0.01^cC^
Phe	0.29 ± 0.02^a^	0.29 ± 0.05^aA^	0.19 ± 0.01^bA^	0.29 ± 0.02^a^	0.29 ± 0.01^aAB^	0.19 ± 0.01^bAB^	0.29 ± 0.02^a^	0.20 ± 0.01^bB^	0.13 ± 0.02^cB^
His	0.12 ± 0.01^a^	0.12 ± 0.00^aA^	0.09 ± 0.02^bA^	0.12 ± 0.01^a^	0.12 ± 0.01^aA^	0.08 ± 0.00^bA^	0.12 ± 0.01^a^	0.10 ± 0.00^aA^	0.05 ± 0.01^bA^
Lys	0.19 ± 0.00^a^	0.19 ± 0.01^aA^	0.10 ± 0.00^bA^	0.19 ± 0.00^a^	0.17 ± 0.01^aA^	0.10 ± 0.02^bA^	0.19 ± 0.00^a^	0.11 ± 0.02^bB^	0.07 ± 0.01^cB^
Arg	0.79 ± 0.04^a^	0.79 ± 0.05^aA^	0.56 ± 0.07^bA^	0.79 ± 0.04^a^	0.72 ± 0.01^aAB^	0.46 ± 0.00^bAB^	0.79 ± 0.04^a^	0.69 ± 0.02^bB^	0.43 ± 0.02^cB^
Pro	0.38 ± 0.00^a^	0.37 ± 0.09^aA^	0.27 ± 0.01^aA^	0.38 ± 0.00^a^	0.37 ± 0.01^aA^	0.20 ± 0.02^bA^	0.38 ± 0.00^a^	0.30 ± 0.01^bA^	0.15 ± 0.00^cA^
EAA	0.89 ± 0.05^a^	0.84 ± 0.03^aA^	0.51 ± 0.02^bA^	0.89 ± 0.05^a^	0.89 ± 0.02^aA^	0.55 ± 0.00^bA^	0.89 ± 0.05^a^	0.61 ± 0.00^bB^	0.39 ± 0.00^cB^
TAA	6.30 ± 0.32^a^	6.30 ± 0.22^aA^	3.99 ± 0.16^bA^	6.30 ± 0.32^a^	4.41 ± 0.00^bB^	2.72 ± 0.04^cB^	6.30 ± 0.32^a^	5.66 ± 0.21^aC^	3.41 ± 0.06^bC^

Note

Data are expressed as mean ± *SD* with three replications. Different lowercase letters for each proximate composition of the same peanut variety and at under same storage temperature indicate statistically significant different values (*p* < 0.05). Different capital letters for each proximate composition of the same peanut variety and at the same storage time indicate statistically significant different values (*p* < 0.05).

Abbreviations: EAA, essential amino acids; TAA, total amino acids.

## 4 CONCLUSIONS

This study showed that storage temperatures (15°C, 25°C, and 35°C) led to different degrees of lipid oxidation of peanuts. The proximate, fatty acid, and amino acid compositions changed significantly during storage which led to the nutrition loss of peanuts. Higher temperatures led to a higher degree of lipid oxidation and nutrients loss. The results provide a reference for the actual storage process of peanuts. Storage at 15°C or short‐term storage at 25°C was suitable for peanuts.

## CONFLICT OF INTEREST

The authors declare that they do not have any conflict of interest.

## ETHICAL APPROVAL

The study does not involve any human or animal testing.
