# Supplementary material for: Effect of storage temperature on lipid oxidation and changes in nutrient contents in peanuts
Source: Food Sci Nutr. 2019 Jun 11;7(7):2280–90. doi: 10.1002/fsn3.1069 (PMC6657719; doi:10.1002/fsn3.1069)
Supplement: Supplementary file 1 [file FSN3-7-2280-s001.pdf]

SUPPLEMENTARY MATERIALS:

Supplementary Table 1.

The information of different fatty acid methyl ester of the **Fig 1.**

Number	Keep time	Peak area	Peak height	Peak width	Symmetry factor	Peak area(%)	Fatty acid
1	9.010	13	7.7	0.0269	0.936	2.34	C4:0
2	9.560	14.7	8.3	0.0287	0.999	2.658	C6:0
3	10.574	16.3	8.1	0.0314	0.999	2.939	C8:0
4	12.319	17.2	7.3	0.0371	1.004	3.109	C10:0
5	13.514	8.7	3.4	0.0404	1.004	1.577	C11:0
6	14.909	18	6.5	0.0434	1.01	3.244	C12:0
7	16.460	9.2	3.1	0.0466	1.006	1.663	C13:0
8	18.116	19	6.2	0.0487	1.005	3.428	C14:0
9	19.410	9.1	2.9	0.0498	1.004	1.637	C14:1
10	19.815	9.2	2.8	0.0515	1.001	1.666	C15:0
11	21.135	9.2	2.8	0.0506	0.987	1.652	C15:1
12	21.523	27.3	8.4	0.0507	1.04	4.934	C16:0
13	22.614	9	2.7	0.0523	1.017	1.616	C16:1
14	23.194	6.6	1.9	0.0535	0.926	1.189	C17:0
15	24.277	9.3	2.8	0.0519	1.009	1.673	C17:1
16	24.824	18.6	5.5	0.0533	0.989	3.364	C18:0
17	25.442	9.2	2.7	0.0531	1.012	1.657	C18:1n9t

18	25.754	18.9	5.6	0.0527	1.019	3.417	C18:1n9c
19	26.423	7.9	2.3	0.0539	1.022	1.433	C18:2n6t
20	27.115	8.8	2.6	0.054	0.991	1.581	C18:2n6c
21	27.906	21.8	6.4	0.0534	1.028	3.939	C20:0
22	28.081	7.9	2.3	0.0533	0.987	1.43	C18:3n6
23	28.660	8.1	2.4	0.0546	1.009	1.465	C20:1
24	28.788	9.6	2.8	0.0538	1.009	1.726	C18:3n3
25	29.353	13.9	4	0.0541	1	2.509	C21:0
26	30.093	8.7	2.5	0.0554	0.98	1.576	C20:2
27	30.774	42.7	12	0.056	1.061	7.701	C22:0
28	31.039	6.3	1.7	0.0564	1	1.135	C20:3n6
29	31.664	6.9	2.3	0.0506	0	1.247	C22:1n9
30	31.767	10.2	2.6	0.0652	1	1.835	C20:3n3
31	32.203	6.7	1.8	0.058	0.988	1.213	C23:0
32	33.055	31.1	8	0.0605	1.06	5.607	C20:4n6
33	33.435	8.7	2.2	0.0623	0.997	1.569	C22:2
34	33.716	5.6	1.3	0.0637	0.997	1.003	C24:0
35	34.732	93	21.5	0.0672	1.292	16.779	C20:5n5
36	36.702	8.7	2	0.068	1.041	1.564	C24:1
37	37.936	5.1	9.90E-01	0.0807	1.046	0.928	C22:6n3

Figure captions:

Supplementary Fig 1. The scheme of the analytical procedures.

Supplementary Fig 2. Gas chromatogram of the standard fatty acid methyl ester.

Supplementary Fig 3. Gas chromatogram of the unstored peanuts (YH-9326) fatty acid composition.

Supplementary Fig 4. Gas chromatogram of peanut (YH-9326) fatty acid composition when stored at 15 °C for 80 d (A), 160 d (B), 240 d (C) and 320 d (D), respectively.

Supplementary Fig 5. Gas chromatogram of peanut (YH-9326) fatty acid composition when stored at 25 °C for 80 d (A), 160 d (B), 240 d (C) and 320 d (D), respectively.

Supplementary Fig 6. Gas chromatogram of peanut (YH-9326) fatty acid composition when stored at 35 °C for 80 d (A), 160 d (B), 240 d (C) and 320 d (D), respectively.

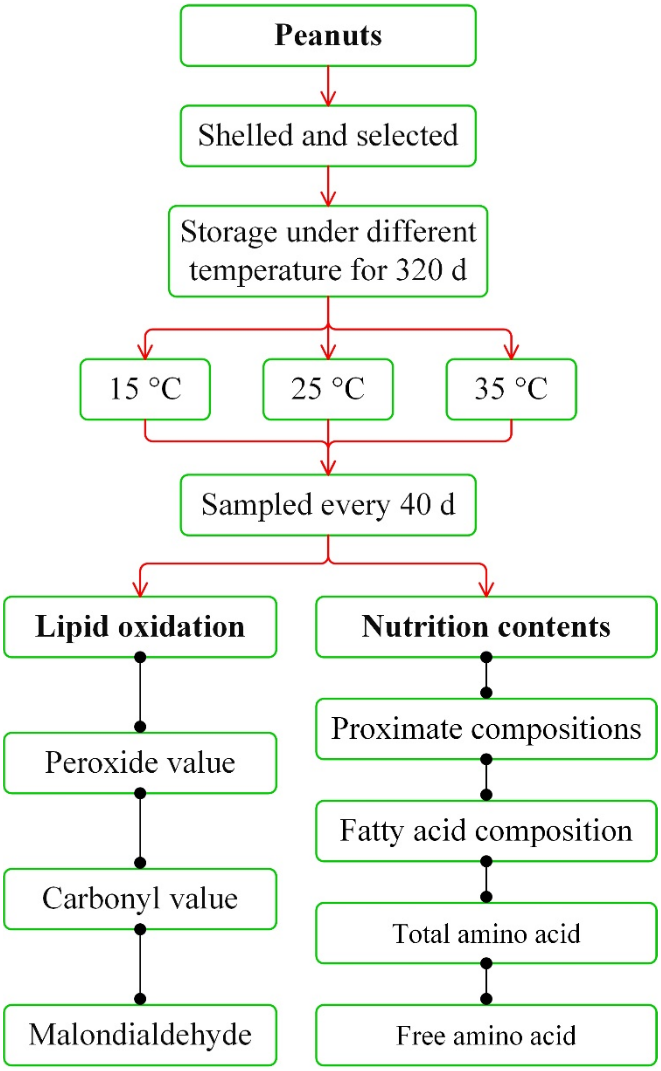

Supplementary Fig 1

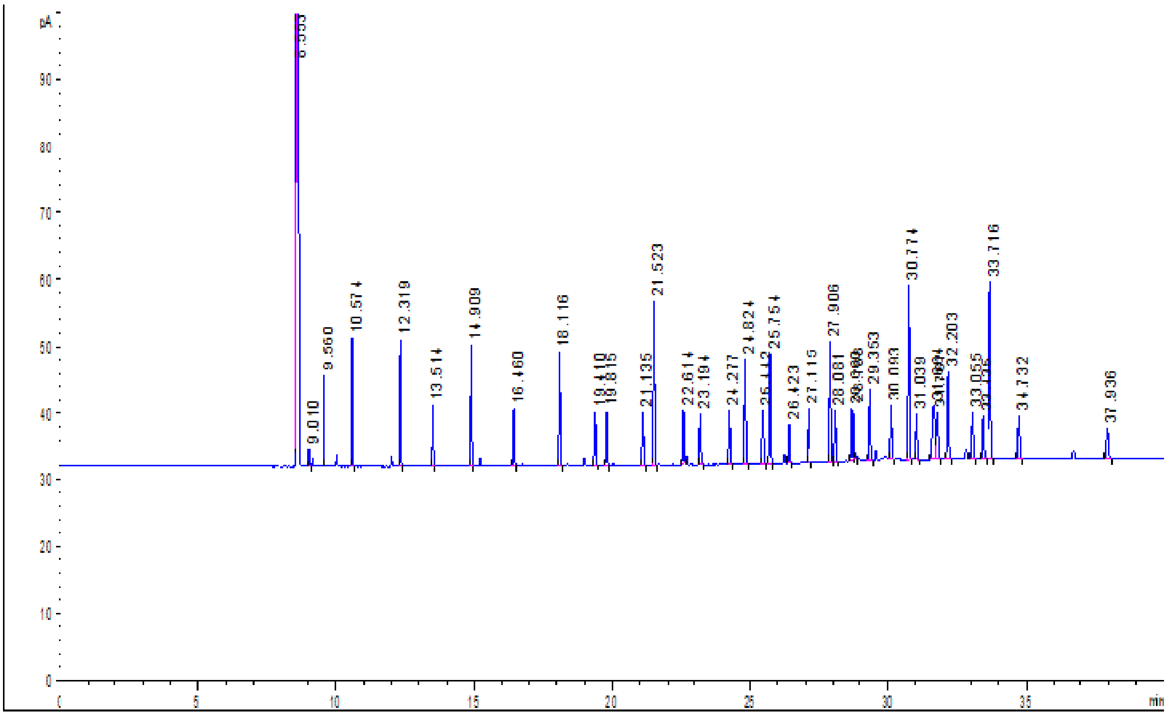

Supplementary Fig. 2

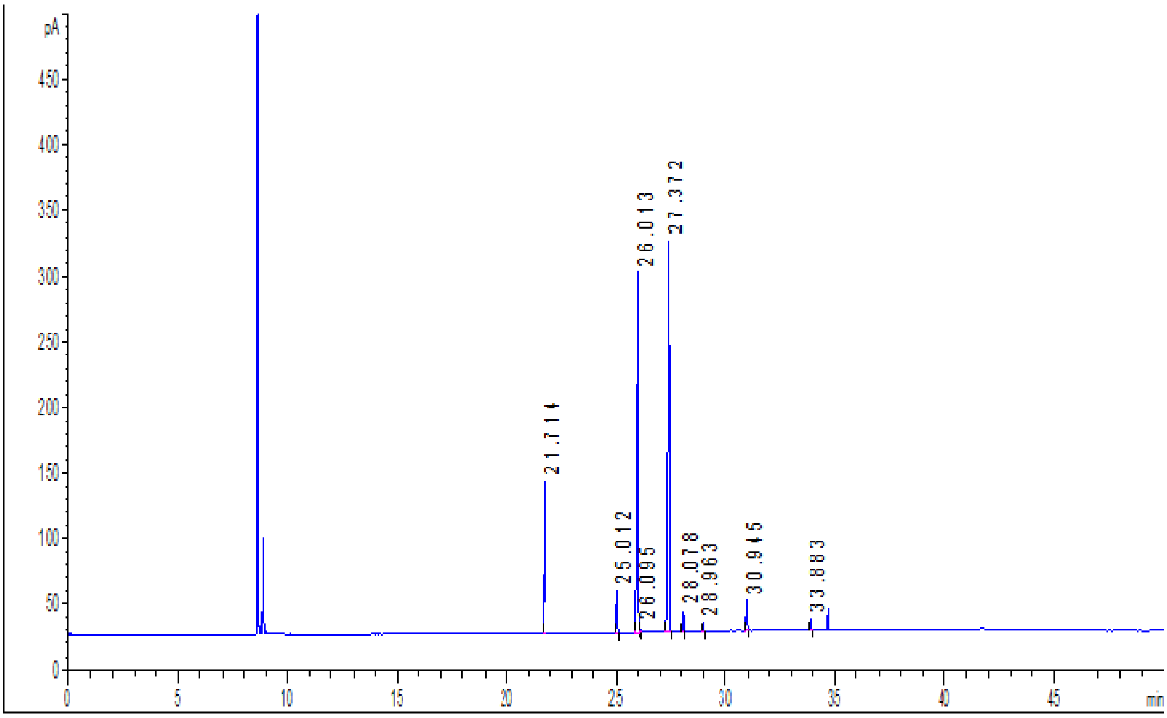

Supplementary Fig. 3

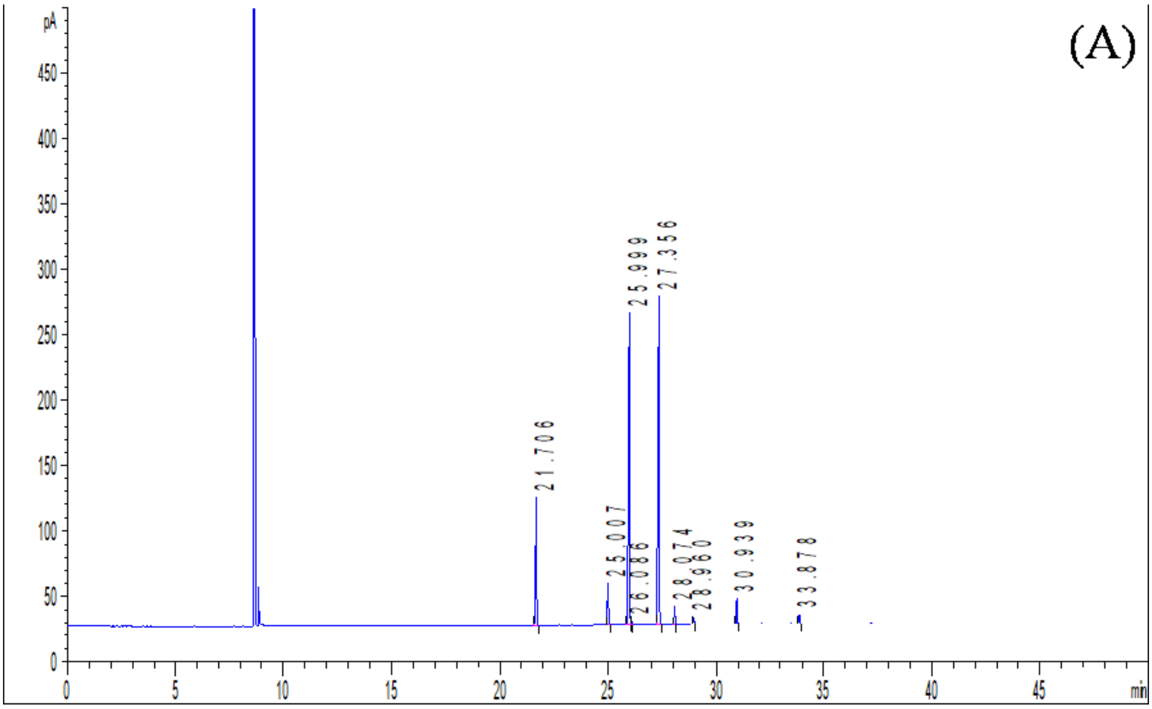
(A)
pA
min
21.706
25.007
25.999
26.086
27.356
28.074
28.960
30.939
33.878

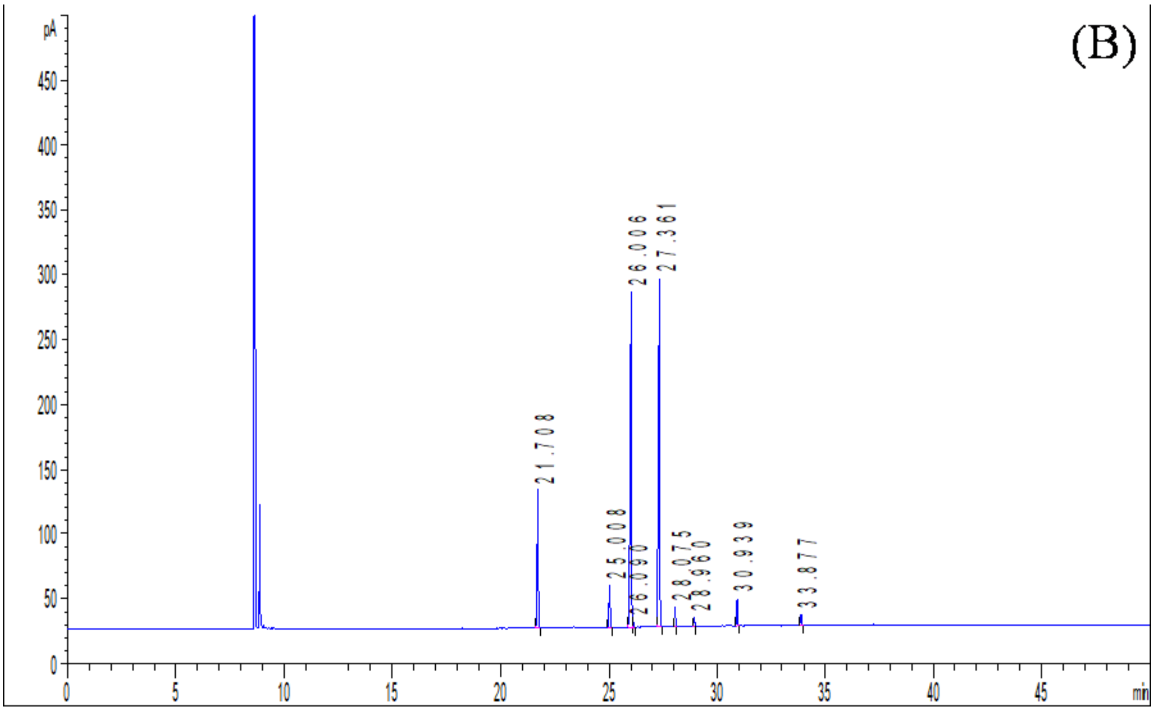
(B)
pA
min
21.708
25.008
26.006
26.090
27.361
28.075
28.960
30.939
33.877

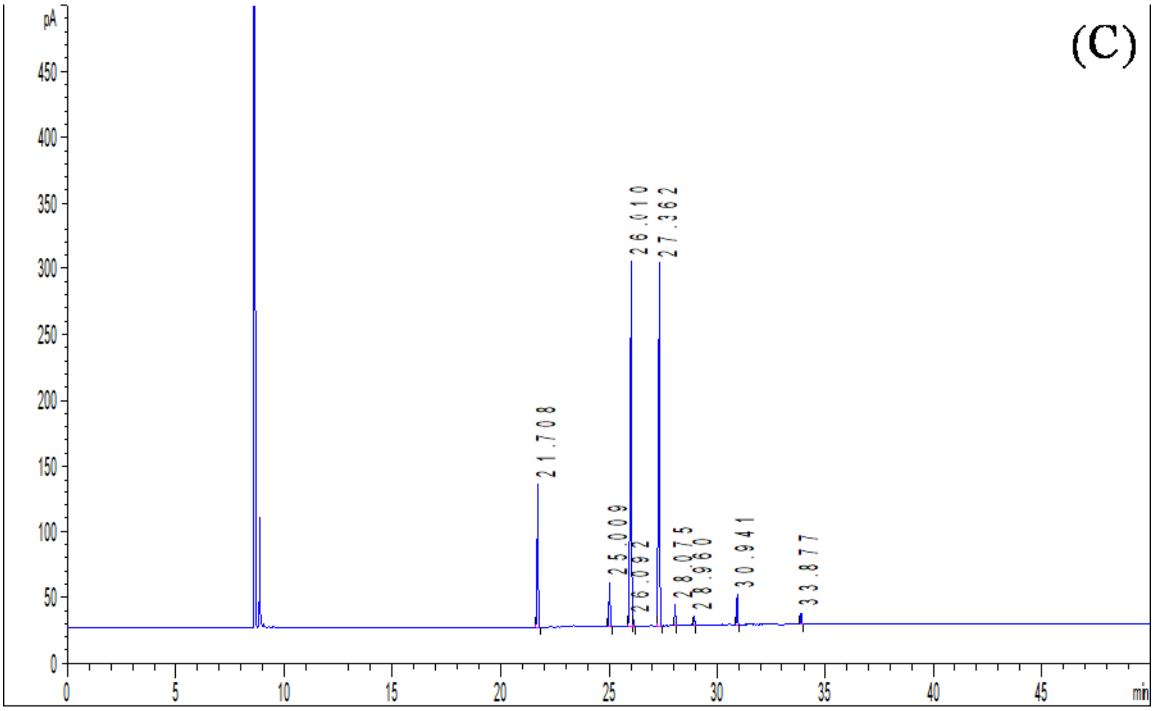

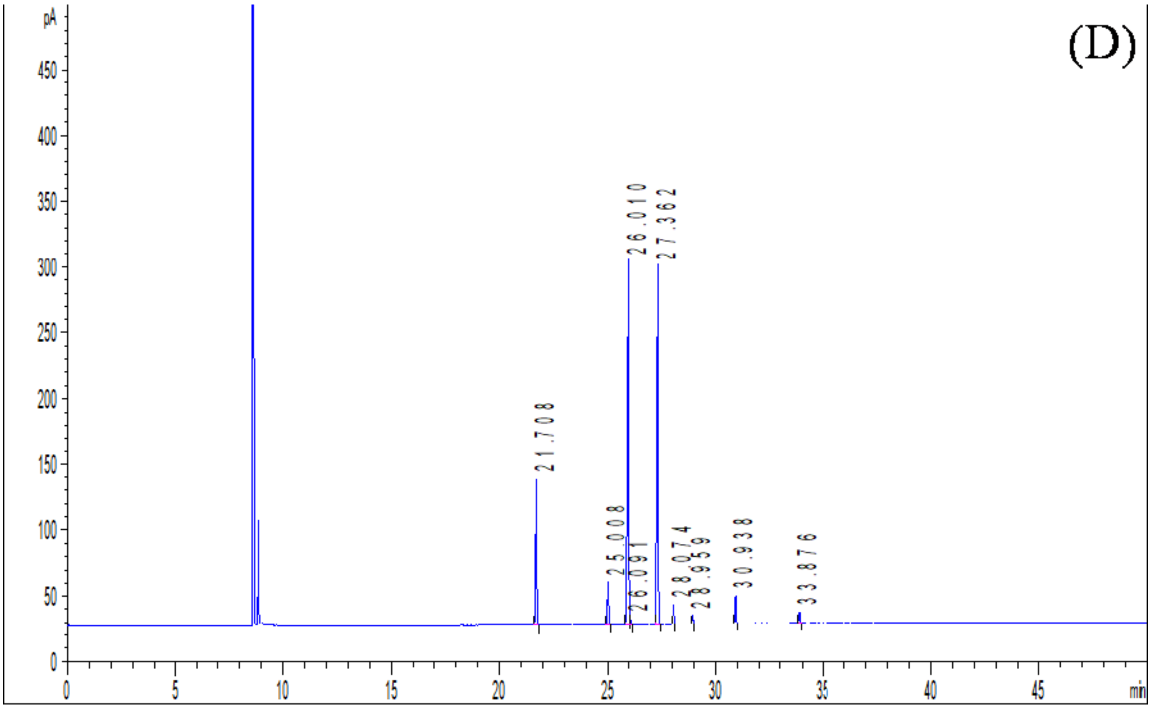

Supplementary Fig. 4

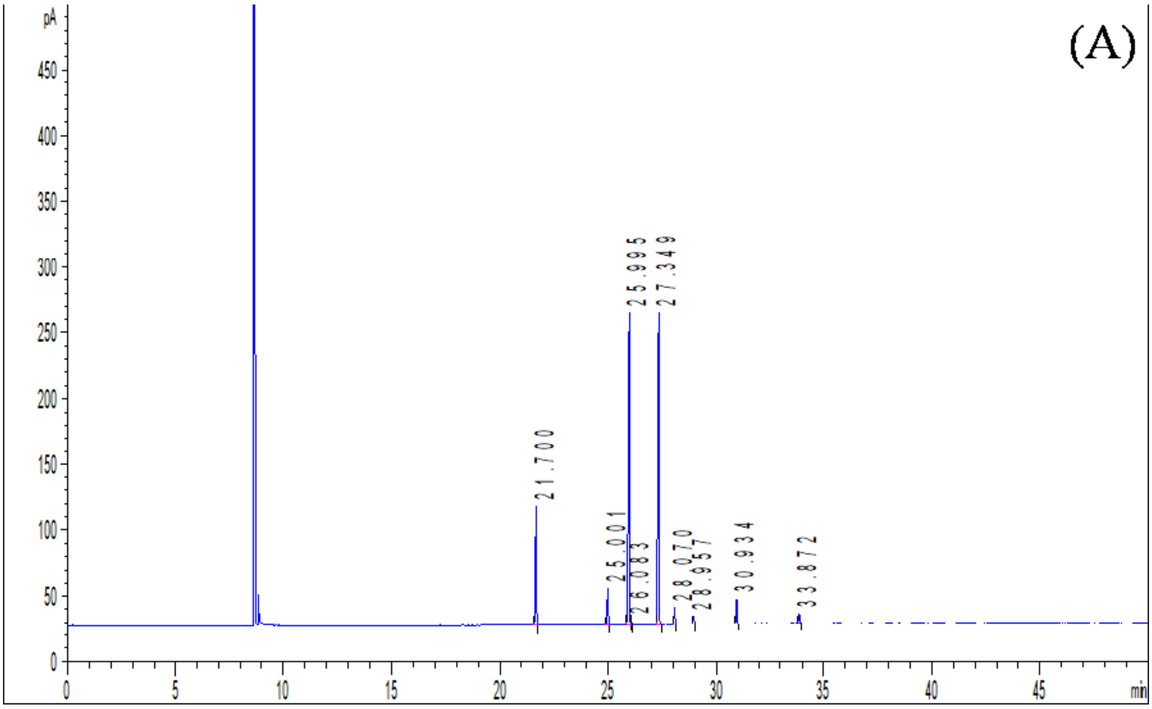
(A)
pA
min
21.700
25.001
25.995
26.083
27.349
28.070
28.957
30.934
33.872

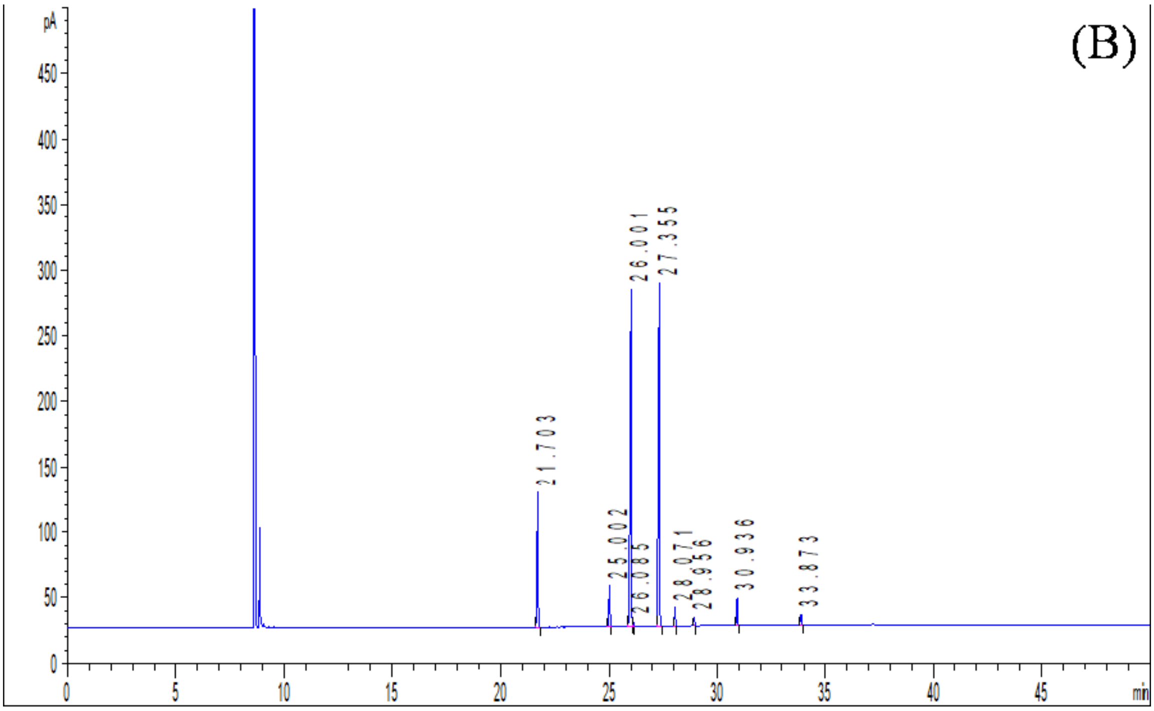
(B)
pA
min
21.703
25.002
26.001
26.085
27.355
28.071
28.956
30.936
33.873

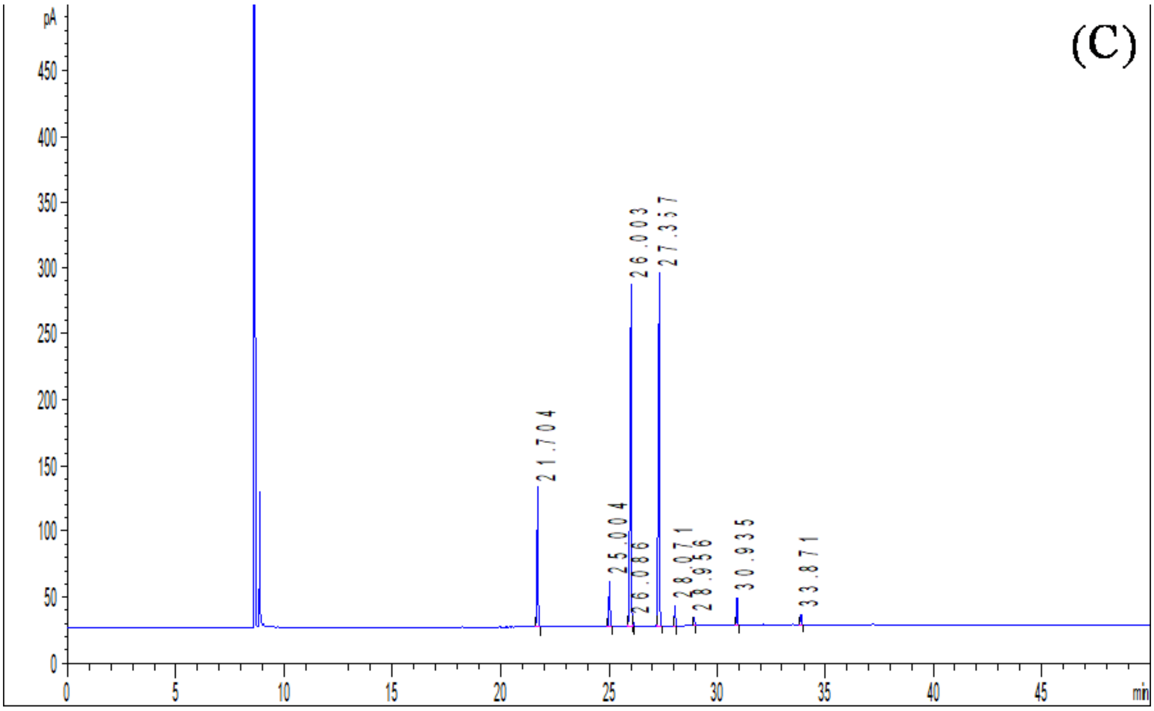

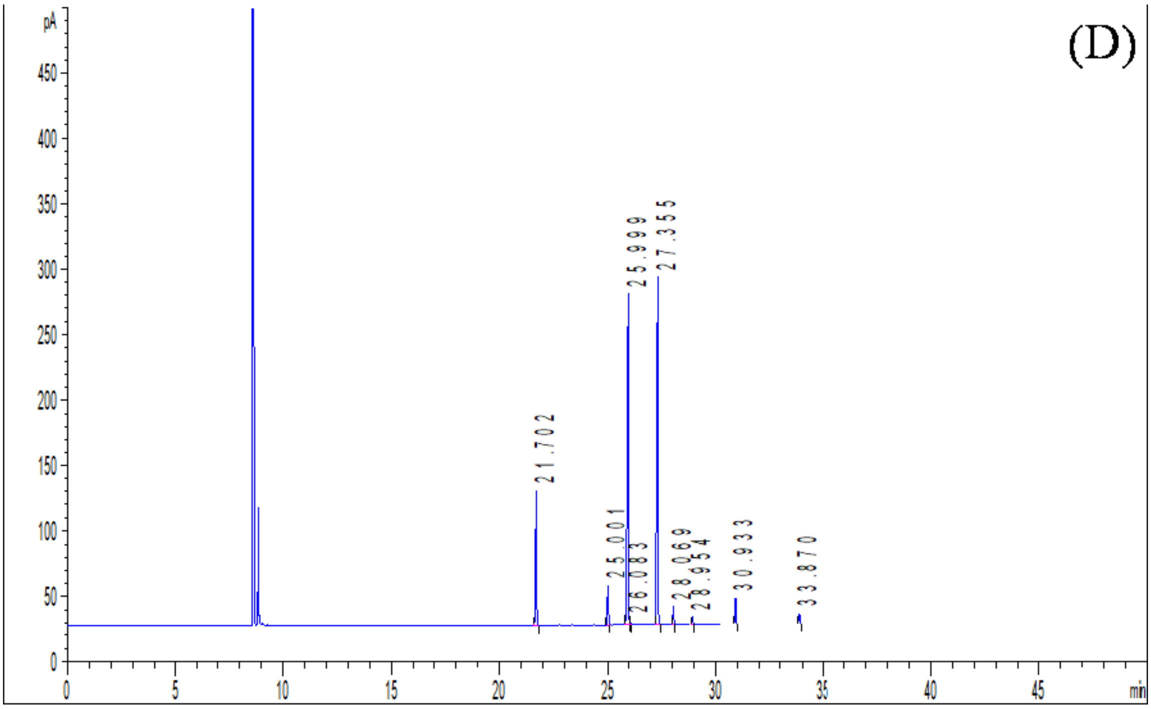

Supplementary Fig. 5

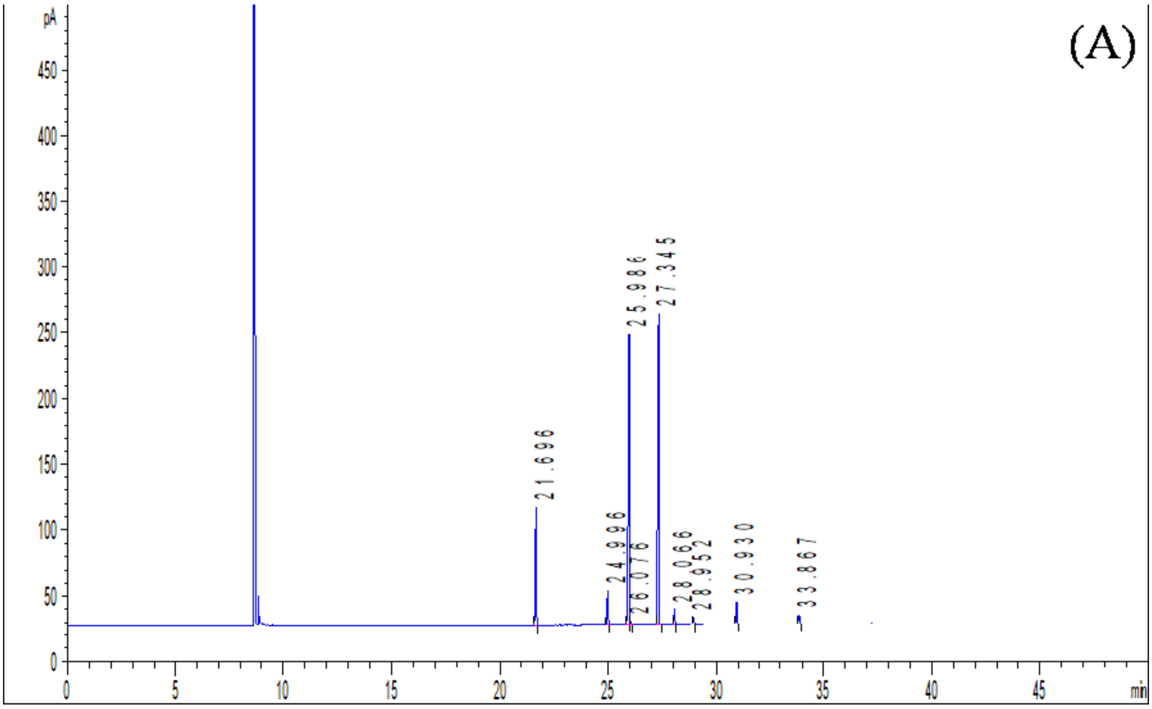
(A)
pA
450
400
350
300
250
200
150
100
50
0
21.696
24.996
25.986
26.076
27.345
28.066
28.952
30.930
33.867
0
5
10
15
20
25
30
35
40
45
min

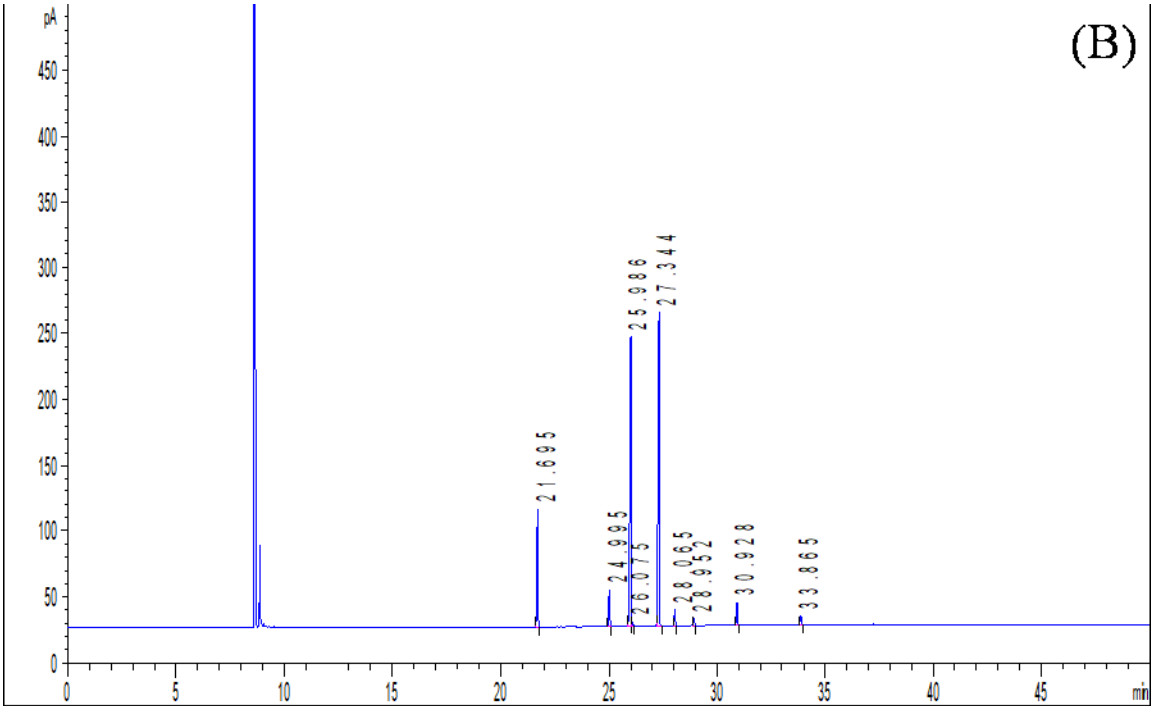
(B)
pA
450
400
350
300
250
200
150
100
50
0
21.695
24.995
25.986
26.075
27.344
28.065
28.952
30.928
33.865
0
5
10
15
20
25
30
35
40
45
min

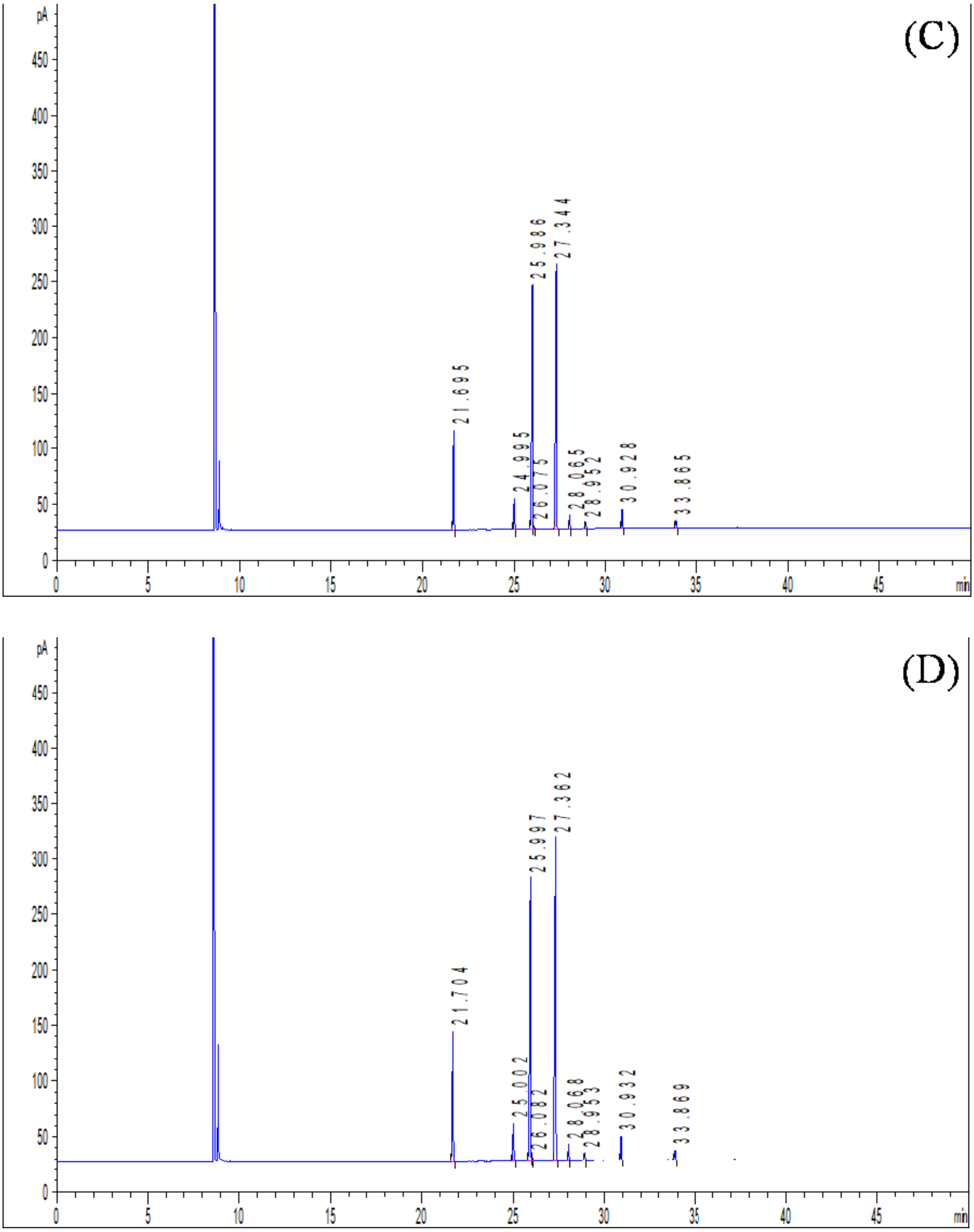

Supplementary Fig. 6
